# Physiologically based pharmacokinetic model combined with a clinical lactation study to determine doravirine concentrations in human breastmilk

**DOI:** 10.1002/bcp.70325

**Published:** 2025-11-06

**Authors:** Lena van der Wekken‐Pas, David Burger, Rick Greupink, Angela Colbers

**Affiliations:** ^1^ Radboud university medical center, department of Pharmacy, Pharmacology and Toxicology Radboud Institute for Medical Innovations (RIMI) Nijmegen the Netherlands

**Keywords:** clinical lactation study, doravirine, HIV, human breastmilk, PBPK model

## Abstract

**Aims:**

To determine breastmilk concentrations of doravirine via PBPK modelling in combination with a clinical lactation study. As women living with HIV are not able to cease antiretroviral therapy during lactation, it is necessary to establish drug transfer into breastmilk to determine exposure of the infant to antiretroviral drugs and potential risk of drug‐induced toxicity.

**Methods:**

PBPK modelling was performed with Simcyp v22 with the lactation module as an add‐on. Model predictions were compared with observed data, and model performance was considered adequate if predicted: observed ratios for AUC_0–24_, C_max_ and half‐life fell between 0.7 and 1.43. Observed data originated from a study performed in healthy, lactating women from whom a full pharmacokinetic curve over 24 h was obtained in both plasma and breastmilk after a single dose of 100 mg doravirine. Concentrations were measured using validated LC–MS/MS assays. Noncompartmental analysis was performed within Phoenix Winnonlin.

**Results:**

The lactation model predicted a geometric mean (CV%) breastmilk to maternal plasma ratio of 0.39,^20^ whereas it was 0.26^21^ in the clinical part of the study. The median (IQR) predicted and observed daily infant dose based on an estimated breastmilk intake of 150 mL/day was 0.18 (0.12–0.29) and 0.18 (0.15–0.26) mg/day. The relative infant dose based on an estimated intake of 150 mL/day was predicted to be 2.27 (1.31–3.94)% and was 1.90 (1.50–2.44) % in the clinical part of the study.

**Conclusions:**

We present in silico and clinical evidence of the transfer of doravirine into breastmilk. However, based on these data, the infant's exposure to doravirine through breastmilk is not expected to be hazardous.

**What is already known about this subject**

- No reports on doravirine in human breastmilk are currently available.
- Such data is needed to inform women living with HIV, as they cannot cease antiretroviral treatment during lactation.

**What this study adds**

- Doravirine does transfer into breastmilk after oral ingestion.
- Breastmilk concentrations of doravirine are low and not expected to cause infant toxicity.
- Combining PBPK modelling simulations with clinical data from healthy volunteers can provide timely lactation information.

## 1 INTRODUCTION

Of the 20 million women who are living with HIV worldwide,^1^ annually, 1.3 million women become pregnant.^2^ According to WHO guidelines,^3^ most women will breastfeed their infants. It is necessary to continue the intake of antiretrovirals (ARV) throughout the full peripartum and breastfeeding period in order to maintain personal health, but also to reduce the risk of transmission through breastmilk. Although it is of utmost importance to prevent this transmission, the possible risk of toxicity due to exposure through breastmilk should also be taken into account.

However, data from clinical trials on breastmilk transfer of ARVs is sparse. Due to (perceived) ethical aspects, such trials are seldom conducted. Some data is being collected prospectively (NCT05648201, NCT05642481),^4^ but a time lag between registration of ARVs and the publication of breastmilk transfer data still exists. This potentially puts infants at unnecessary risk for toxicity while awaiting results from such clinical studies.

For doravirine, a non‐nucleoside reverse transcriptase inhibitor, no human data on breast milk transfer are available yet, whereas the drug was already approved for adults by the FDA in 2018. Animal studies show transfer of doravirine into breastmilk,^1^, ^5^ but these models do not necessarily represent the human situation adequately due to species differences in the mechanisms that determine milk excretion.^2^, ^6^ Doravirine is a first‐line ARV, and it is being used increasingly, for example, to avoid weight gain or neuropsychiatric side effects that are attributed to the often‐used integrase strand transfer inhibitors. Also, the implementation of this drug in low‐ and middle‐income countries will increase its use. In these regions of the world, breastfeeding is the norm, also in the case of an existing HIV infection.^3^ This further underscores the need for sufficient data on breastmilk transfer of this drug.

Physiologically based pharmacokinetic (PBPK) modelling can help to estimate breastmilk transfer and subsequent infant exposure. PBPK models are mathematical models based on physicochemical and in vitro pharmacokinetic properties of a drug, as well as the anatomical, physiological and biochemical characteristics of a virtual target population of interest, that allow to simulate drug exposure in plasma and tissues, as well as breastmilk.^3^, ^7^, ^8^ This type of models have been accepted to inform drug development, dose selection in first in‐human trials, predict drug–drug interactions and simulate drug behaviour in different populations.^9^ The aim of the current study is to determine breastmilk concentrations of doravirine by combining simulations of such a PBPK model with data derived from a clinical study in healthy, lactating volunteers without HIV.

## 2 METHODS

In order to determine doravirine concentrations in human breastmilk, Simcyp PBPK Simulator version 22 (Certara company, Sheffield, UK) was used to build the PBPK model and perform simulations. The simulated concentrations and pharmacokinetic (PK) parameters were subsequently compared to data derived from a clinical study in healthy, lactating volunteers. A visual overview of the study is provided in the supplementary material (Figure S1).

### 2.1 Validation of the model in a non‐lactating population

To construct the PBPK model, a previously published and validated model^10^, ^11^ for doravirine was used (compound file in supplementary data). First, this compound file was validated using previously published PK data from clinical studies in non‐lactating populations (search query and summary of literature findings in supplementary file). Data from time‐concentration curves from these clinical studies (both single and multiple oral dose studies) were extracted using Automeris Webplotdigitizer version 4.5. For each clinical study, 10 trials were run for the exact number of participants in the clinical studies, using matched demographic values.

We chose to run a minimal PBPK model because no organ‐specific transporters are described for doravirine, and no high partitioning in specific tissues is expected. Because no food interaction is to be expected for doravirine^12^ and only one formulation is currently under research, we chose a first‐order absorption model instead of ADAM/M‐ADAM (advanced dissolution, absorption and metabolism/multilayer gut wall within ADAM) and used a fasted state. Model predictions were compared with observed data, and model performance was considered adequate if predicted: observed ratios for AUC_0–24_, C_max_ and half‐life fell within strict criteria (between 0.7 and 1.43), to ensure proper confidence in the model.^13^ As data i on variability of doravirine concentrations in breastmilk is lacking, strict criteria were selected.

### 2.2 Lactation model

After the compound file was validated, it was combined with an add‐on lactation module, which is present in the Simcyp® PBPK software package.^7^ With this model, breastmilk: maternal plasma ratio was predicted based on maternal plasma and breastmilk pH, protein binding and partitioning of the compound into fat in breastmilk, using the phase distribution model.^14^, ^15^ As doravirine is a monoprotic base, with a logP of 3.0 and moderately protein bound, and is not a known substrate for transporters, we believe parameters used in aforementioned model are sufficient to describe breastmilk: maternal plasma ratio and a perfusion limited model would be sufficient. Lastly, the model was set to dynamic, which allowed for an increase in breastmilk pH in the course of time post‐partum, which resembles the physiologic situation.^16^

Ultimately, 10 simulation trials within 10 subjects were performed. Demographic data of the virtual population was matched (age, weight and post‐partum age) with those of the participants of the clinical part of the study (see below). Predicted concentrations and PK parameters were compared to those from the clinical part of the study with the use of visual predictive plots and by tabulation of ratios of area under the curve (AUC_inf_), maximum concentration (C_max_), half‐life (T_½_) using aforementioned strict criteria (between 0.7 and 1.43). Also, the calculated breastmilk: maternal plasma ratio, the daily infant dose and relative infant dose were estimated with the use of the formulas below.^17^ As volumes of expressed milk may vary among study participants, an estimation with a daily intake of 150 mL/kg^18^ of infant weight will also be reported to better allow generalization.

- Breastmilk: maternal plasma ratio = AUC_breastmilk_/AUC_maternal plasma_.
- Daily infant dosage (mg/day) = Σ (total drug concentration in each milk collection multiplied by the expressed milk volume in each milk collection).
- Daily infant dosage (mg/day) with estimated milk intake of 150 mL/kg/day = (AUC_breastmilk_/24) * (150*weight of infant).
- Relative infant dose (%) = Infant Dosage (mg/kg/day)/Maternal Dosage (mg/kg/day) multiplied by 100.

### 2.3 Clinical part of the study

The clinical part of the study was preregistered (NCT05648201) and conducted in accordance with the principles of the Declaration of Helsinki (8th version, 2013), Declaration of Tapei (2016) and in accordance with the Medical Research Involving Human Participants Act (WMO). The medical ethical committee of East Netherlands approved the study (NL83180.091.22). Healthy, lactating volunteers were recruited for this study who were willing and able to withhold from breastfeeding their infants up till 4 days after study participation. To prevent unnecessary exposure of breastfed infants, participants were eligible when they wanted to stop breastfeeding altogether, or if they could temporarily provide alternative feeding, while keeping up milk production using a pump and discarding the breastmilk potentially containing the study drug up till 4 days after ingestion of doravirine. Assuming breastmilk concentrations are dependent on plasma concentrations, and the plasma half‐life of doravirine is 15 h, a period of 4 days was deemed sufficient. Exclusion criteria were the presence of a severe galactose intolerance, HIV infection or co‐morbidity of use of co‐medication known to interfere with the pharmacokinetics of doravirine. Written informed consent was obtained before study interventions took place.

After a single oral dose of 100 mg of doravirine was administered, plasma samples were collected at t = 0, 1, 2, 3, 4, 5, 6, 8, 10, 12, 24 h post ingestion and breastmilk samples were collected at t = 0, 2, 4, 6, 12, 24 h after dosing. Participants were instructed to use an electric pump and to empty both breasts at every predefined time point. The obtained breastmilk from each timepoint was mixed and aliquoted. The timing and volume of each sample were noted.

Drug concentrations in plasma were measured using an in‐house developed and externally validated^19^, ^20^ assay using liquid chromatography with tandem mass spectrometry (LC–MS/MS). Drug concentrations in breastmilk were measured using an in‐house developed LC–MS/MS assay,^21^ internally validated in breastmilk according to EMA guidelines.^22^ The lowest level of quantification (LLOQ) for doravirine in plasma and breastmilk was 0.01 mg/L. Phoenix Winnonlin® (Certara company, Sheffield, UK) was used to perform noncompartmental analysis to determine pharmacokinetic parameters in plasma and breastmilk from these measured concentrations.

Ultimately, simulations of the PBPK model with multiple doses (100 mg once daily during 5 days) and the M/P ratio observed in the clinical part of the study were performed to predict infant exposure to doravirine through breastmilk.

## 3 RESULTS

### 3.1 Outcomes of PBPK model validation and lactation simulations

Details of the model validation in a non‐lactating population are summarized in supplementary material (compound file (Table S1), search query, overview of studies in non‐lactating population (Table S2)). The predicted and observed PK parameters in a non‐lactating population are depicted in Table 1. The predicted observed ratios met the predetermined criteria, except for the C_max_ in one study.^23^

**TABLE 1:** Predicted and observed geometric means (95% CI or CV%) of doravirine plasma PK parameters in non‐lactating populations, as well as derived ratio's.

Single dose PK studies (100 mg)	Predicted	Observed	Ratio
AUC_inf_ (mg/L*h)	19.4 (17.4–21.8))	19.2 (14.1–26.1)^23^	1.01
		23.25 (17.9–30.23)^24^	0.83
		16.65 (13.4–20.7)^11^	1.17
C_max_ (mg/L)	0.90 (0.83–0.96)	0.81 (0.62–1.06)^23^	1.11
		0.87 (0.67–1.14)^24^	1.03
		0.89 (0.77–1.03)^11^	1.01
T_1/2_ (h)	13.06 (43)	16.7 (26.1)^23^	0.75
		18.1 (30.5)^24^	0.72
		13.8 (31.9)^11^	0.95
Multiple dose PK studies			
AUC_tau_ (mg/L*h)	18.38 (16.4–20.57)	17.59 (15.12–20.48)^25^	1.04
		15.46 (12.86–18.61)^23^	1.19
		14.48 (11.79–17.76)^26^	1.27
C_max_ (mg/L)	1.25 (1.14–1.36)	1.45 (1.26–1.66)^25^	0.86
		0.71 (0.60–0.85)^23^	1.76
		1.04 (34)	1.20
T_1/2_ (h)	12.9 (48)	NA^25^	NA
		14.3 (25.6)^23^	0.90
		NA^26^	NA

Results of simulations of the lactation model are summarized in Table 2. All predicted PK parameters, RID and DID fell within predefined criteria, except for the breastmilk: maternal plasma ratio, which was overpredicted.

**TABLE 2:** Predicted and observed geometric mean (CV%) and median (IQR) doravirine PK parameters in plasma and breastmilk by PBPK lactation model.

PK parameters	Predicted n = 100	Observed n = 8	Ratio
*Demographics*	Median (range)	Median (range)	
Age (years)	33.9 (28.3–39)	32 (28–39)	1.06
Weight (kg)	65.8 (42.5–106.1)	80.8 (60.6–140)	0.81
Post partum age (months)	7.8 (2.0–12)	6.5 (2–38)	1.2

*Plasma*	GM (CV%)	GM (CV%)	
AUC_inf_ (mg/L*h)	16.70 (56)	20.75 (30)	0.80
C_max_ (mg/L)	1.03 (28)	1.19 (36)	0.87
T_1/2_ (h)	9.43 (49)	13.17 (33)	0.72

*Breastmilk*	GM (CV%) or median (IQR)	GM (CV%) or median (IQR)	
Calculated milk: plasma ratio	0.39 (20)	0.26 (21)	1.50
AUC_inf_ (mg/L*h)	6.55 (54)	5.31 (24)	1.23
C_max_ (mg/L)	0.40 (36)	0.31 (33)	1.29
T_1/2_ (h)	9.43 (49)	11.56 (34)	0.82
Daily infant dose (mg/day)		0.07 (0.01–0.11)	
Relative infant dose (%)		0.86 (0.08–1.42)	
Daily infant dose (mg/day) based on estimated intake of 150 mL/kg of infant weight	0.18 (0.12–0.29)	0.18 (0.15–0.26)	1
Relative infant dose (%) based on estimated intake of 150 mL/kg of infant weight	2.27 (1.31–3.94)	1.90 (1.50–2.44)	1.19

### 3.2 Outcomes of clinical part of study

Eight women participated in the study. Their characteristics are summarized in Table 2. They had a median (range) age of 32^27^, ^28^, ^29^, ^30^, ^31^, ^32^, ^33^, ^34^, ^35^, ^36^, ^37^, ^38^ years and weight of 80.8 (60.6–140) kg. The number of months post‐partum varied among participants, with a median (range) of 6.5.^2^, ^3^, ^4^, ^5^, ^6^, ^7^, ^8^, ^9^, ^10^, ^11^, ^12^, ^13^, ^14^, ^15^, ^16^, ^17^, ^18^, ^19^, ^20^, ^21^, ^22^, ^23^, ^24^, ^25^, ^26^, ^27^, ^28^, ^29^, ^30^, ^31^, ^32^, ^33^, ^34^, ^35^, ^36^, ^37^ The gestational age at delivery was (median (range)) 40.5 (38–42) weeks.

**FIGURE 1:**
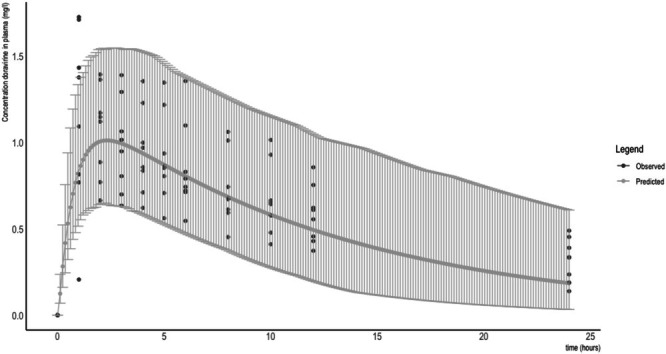
Predicted and observed concentrations of doravirine in plasma after a single oral dose of 100 mg. Data represent the mean with associated 5th and 95th percentiles representing the predicted variability in exposure.

**FIGURE 2:**
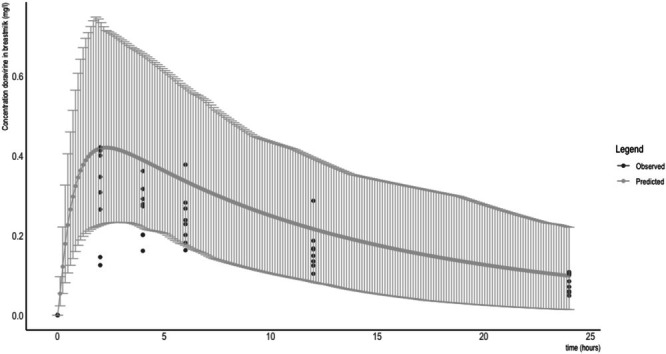
Predicted and observed concentrations of doravirine in breastmilk (mean with 5th and 95th percentile) after a single oral dose of 100 mg. Data represent the mean with associated 5th and 95th percentiles representing the predicted variability in exposure.

Concentrations in both plasma and breastmilk at each time point are depicted in Figures 1 and 2, respectively. No levels fell below the LLOQ. The geometric mean (CV%) AUC_inf_, C_max_ and T_1/2_ in plasma were 20.75^29^ mg/L*h, 1.19^35^ mg/L and 13.17^32^ h, respectively. In breastmilk, these parameters were 5.31^24^ mg/L*h, 0.31^32^ mg/L and 11.56^33^ h, respectively. This resulted in a geometric mean (CV%) breastmilk to maternal plasma ratio of 0.26.^21^ The median (IQR) daily infant dose based on actual volumes expressed by the participants was 0.07 (0.01–0.11) mg/day, while this was 0.18 (0.15–0.26) mg/day when it was calculated assuming a daily infant breastmilk intake of 150 mL/kg. The median (IQR) relative infant dose based on the same assumption was 1.90 (1.50–2.44) %.

**FIGURE 3:**
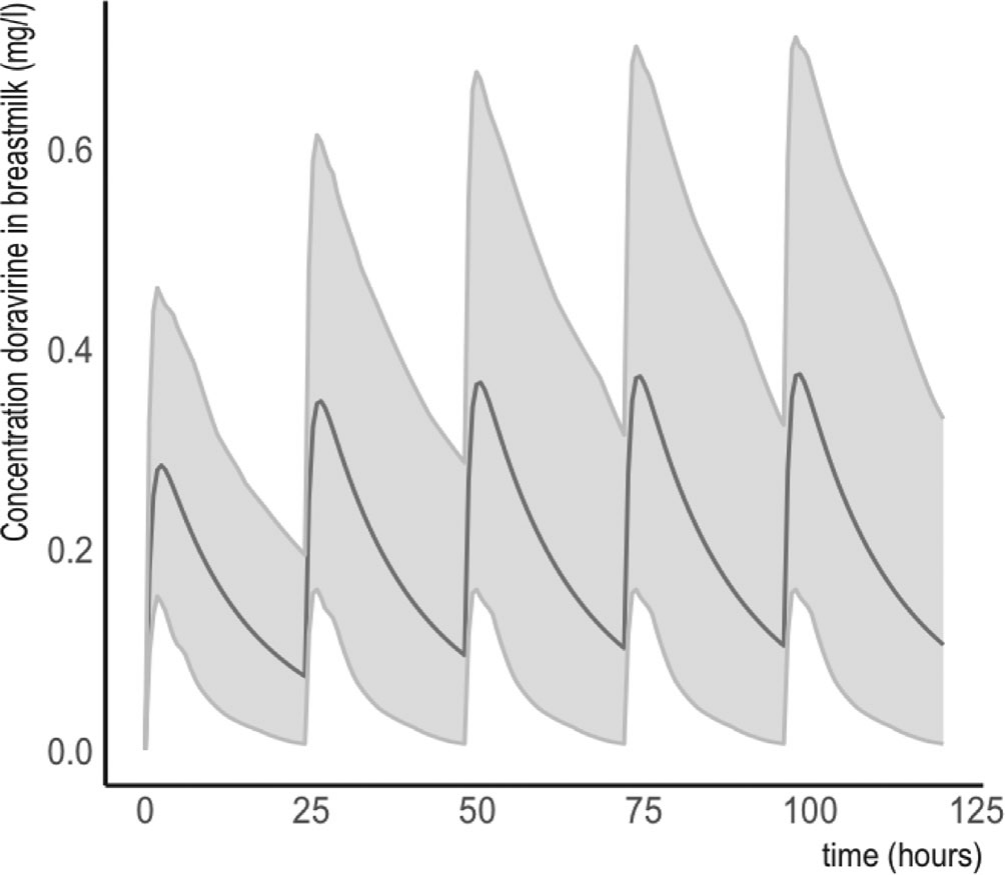
Simulations of mean concentrations and 5th and 95th percentile of doravirine in breastmilk after 100 mg once daily during 5 days.

Outcomes of the simulations with multiple doses and the M/P ratio observed in the clinical part of the study are presented in Figure 3 and Table 3. To illustrate possible changes due to variable compositions of breastmilk, additional sensitivity analyses with a range of breastmilk pH and creamatocrite values were simulated (Table S4A,B).

**TABLE 3:** Simulations with multiple doses and M/P ratio according to observed data from the clinical part of the study.

PK parameters	Predicted n = 100
*Demographics*	Median (range)
Age (years)	32.3 (18.8–45)
Weight (kg)	65.7 (42.5–105.9)
Post partum age (months)	6.8 (0.03–11.9)

*Plasma*	GM (CV%)
AUC (mg/L*h)	17.1 (57)
C_max_ (mg/L)	1.3 (36)
T_1/2_ (h)	9.6 (49)

*Breastmilk*	GM (CV%)
AUC (mg/L*h)	4.4 (69)
C_max_ (mg/L)	0.3 (48)
T_1/2_ (h)	9.6 (49)
Daily infant dose (mg/day) based on estimated intake of 150 mL/kg of infant weight	0.1 (69)
Relative infant dose (%) based on estimated intake of 150 mL/kg of infant weight	1.9 (69)

## 4 DISCUSSION

This study uses an innovative approach to determine doravirine transfer into breastmilk using a combination of in silico predictions and in vivo measurements. As the PBPK model met predefined criteria, it allowed for a smaller sample size in the subsequent clinical study. This approach might be particularly useful in a population where recruitment can be challenging. The inclusion of lactating women who are willing and able to (temporarily) cease breastfeeding to participate in this study has been challenging. As conducting a clinical study is a time‐consuming procedure, this alternative approach allows for the timely publication of important lactation data. Up till now, such data is published (if at all) long after approval of most drugs. This lack of evidence hampers clinical guidance during decision‐making on whether breastfeeding is possible in combination with certain drugs, leaving both clinicians and patients in uncertainty. In some cases, breastfeeding is discouraged altogether because of the paucity of reliable lactation data for certain drugs.

For doravirine, current findings suggest that breastfeeding is safe during its use, even though certain questions cannot be readily answered. Doravirine transfers into breastmilk with a breastmilk: maternal plasma ratio reflecting the unbound fraction of doravirine, which suggests that passive transport is the main transfer mechanism. The estimated daily infant dose and relative infant dose are low. And even though no clear concentration–toxicity relationship has been established, and no specific safety threshold has been determined for doravirine, an RID <5–10% has been proposed as cut‐off in risk assessment of drugs in breastmilk. Indeed, Verstegen 2022 et al^24^ reported that most breastfeeding‐related adverse drug events occur in cases of much higher RIDs. However, several other factors should be considered as well. For instance, not all toxicity is dose‐dependent. Even in smaller doses, adverse drug reactions may occur. However, this type of reaction is rare for doravirine in adults and, therefore, probably in infants as well. Another important factor to consider is the cumulative exposure. As antiretroviral drugs are taken continuously, exposure persists throughout the entire breastfeeding period. Finally, accumulation of even small doses might occur in case of low clearance in the breastfed infant. As doravirine is mainly metabolized by CYP3A4, an enzyme that is not yet at its full capacity in the first 6 months of an infant's life, this might play a role in the ultimate exposure. Besides toxicity, the potential for the development of resistance in case of transmission and subtherapeutic concentrations in breastmilk should be considered. Even though all these factors play an important role, it is not expected that with the low breastmilk concentrations seen in this study, combined with the very low risk of transmission, it will lead to clinically significant exposure in infants. Application of current data in a paediatric PBPK model could be used to verify this.

The PBPK model used in this study was able to correctly predict breastmilk concentrations of doravirine; however, improvements to the current lactation model could further enhance its performance. Currently, pregnancy‐induced pharmacokinetic changes are not yet incorporated. As Bukkems et al (25) showed, the pregnancy‐related induction of CYP3A4 leads to a significant decrease in doravirine concentrations in the third trimester. This might influence infant exposure as soon as breastfeeding is initiated.^25^ In accordance with current recommendations^26^ this study only focused on doravirine concentrations from one month postpartum and onwards, reducing the risk of bias introduced by these pregnancy‐induced pharmacokinetic alterations. Another possible improvement lies in the fact that the current lactation model assumes the same half‐life in plasma as in breastmilk. Yet, the breasts can be regarded as an individual compartment which is emptied every 2 to 6 h when the infant is fed. This might influence the time which is needed to reach a new equilibrium with the plasma compartment and therefore the half‐life. Nonetheless, as model performance is shown to be accurate, further refinement and application in other scenarios can be encouraged.

Some limitations are worth mentioning for this study. First, within Simcyp – the software used to develop and run the current PBPK model – it is not possible to extend simulations in a lactating population beyond 12 months postpartum due to a lack of data. As some participants of the clinical part of this study were breastfeeding for longer than this period, this might have introduced some bias. However, relevant breastmilk composition changes (in terms of pH, fat and protein content) are most profound during the first month of lactation,^27^, ^28^, ^29^ where colostrum transitions into mature milk.^16^ These changes have been accounted for in the dynamic lactation model. Also, we have conducted a sensitivity analysis with the clinical data from only those women who were less than 12 months postpartum, and their parameters did not significantly differ from those who were breastfeeding longer (Table S3 in supplementary material).

A limitation in the clinical part of the study is its single‐dose design, which leaves the possibility for higher concentrations when steady state is reached. However, it is unlikely that – with similar PK parameters in our participants compared to those seen in previous studies (Table S2) and low concentrations in breastmilk – clinically significant higher concentrations will be reached at steady state. This has also been acknowledged by the FDA, which discourages unnecessary exposition to study drugs in lactating participants.^17^ Simulations performed with multiple doses and the M/P ratio observed in the clinical part of the study also point towards low exposure, even after prolonged ingestion of doravirine. Furthermore, it is unknown whether simulations in a healthy population can be extrapolated to a population of women living with HIV. However, as breastfeeding is only recommended in women who have a suppressed plasma viral load, we believe that their pharmacokinetic properties and breastmilk composition will not differ significantly from women who do not live with HIV. Finally, as this study only provides PK data, research on clinical endpoints is recommended. The establishment of an international registry in which adverse drug reactions after ingestion of drugs through breastmilk could provide helpful insights in addition to current PK data, in order to better understand their interplay.

## 5 CONCLUSION

Using an innovative approach in which a PBPK model was combined with clinical data, data on doravirine concentration in breastmilk were generated. Breastmilk: maternal plasma ratio was 0.26, and daily‐ and relative infant dosages were low (0.18 mg/day and 1.9%, respectively). Therefore, the risk of infant toxicity after exposure to doravirine through breastmilk is probably low.

## AUTHOR CONTRIBUTIONS

AC conceived the idea of the study, applied for funding, supervised design and conduct of study, supervised data analysis and revising the manuscript. DB supervised conduct of study and manuscript writing, RG supervised the parameterization of the PBPK model and conduct of simulation and revising the manuscript. LW designed the study, recruited and screened participants, performed data analysis, designed PBPK model and ran simulations, wrote manuscript.

The authors confirm that the PI for this paper is Lena van der Wekken‐Pas and that she had direct clinical responsibility for patients.

## CONFLICT OF INTEREST STATEMENT

LWP has no conflict of interest to declare. AC received research grants from ViiV Healthcare, Gilead, Merck, all paid to the institution. RG received research grants from Unilever and Bill and Melinda Gates foundation, all paid to the institution. DB received research grants from ViiV Healthcare, Gilead, Merck, Pfizer and Takeda, all paid to the institution.

## Supporting information


**Figure S1** General workflow for constructing and validating the PBPK model and predicting exposure through breastmilk using the lactation module.
Table S1 Compound file.
Table S2 Overview of doravirine studies to validate the previously published PBPK model.
Table S3 Sensitivity analysis with data from participants who were shorter or longer than 12 months postpartum.
Table S4A Sensitivity analysis with ranging breastmilk pH; geometric mean (CV%) of pharmacokinetic parameters in breastmilk and DID (mg) and RID.
Table S4B Sensitivity analysis with ranging breastmilk creamatocrite values.

## Data Availability

The data that support the findings of this study are available on request from the corresponding author. The data are not publicly available due to privacy or ethical restrictions.
