# Supplementary material for: Physiologically based pharmacokinetic model combined with a clinical lactation study to determine doravirine concentrations in human breastmilk
Source: Br J Clin Pharmacol. 2025 Nov 6;92(3):952–9. doi: 10.1002/bcp.70325 (PMC12930008; doi:10.1002/bcp.70325)
Supplement: Supplementary file 1 — Figure S1 General workflow for constructing and validating the PBPK model and predicting exposure through breastmilk using the lactation module. Table S1 Compound file. Table S2 Overview of doravirine studies to validate the previously published PBPK model. Table S3 Sensitivity analysis with data from participants who were shorter or longer than 12 months postpartum. Table S4A Sensitivity analysis with ranging breastmilk pH; geometric mean (CV%) of pharmacokinetic parameters in breastmilk and DID (mg) and RID. Table S4B Sensitivity analysis with ranging breastmilk creamatocrite values. [file BCP-92-952-s001.docx]

**Supplementary data**

*Figure S1 General workflow for constructing and validating PBPK model and predict exposure through breastmilk using lactation module*

**Table S1 Compound file**

|  | **Input** | **Reference** |
| --- | --- | --- |
| Molecular weight (g/mol) | 425.75 | Yee et al, 2021 (10) |
| LogP | 3.0 | Yee et al, 2021 |
| Compound type | Monoprotic base | Yee et al, 2021 |
| pKa | 9.47 | Yee et al, 2021 |
| Plasma free fraction | 0.24 | Yee et al, 2021 |
| Blood/plasma ratio | 1.0 | Yee et al, 2021 |
| Intestinal free fraction | 1.0 | Assumed, sensitivity analysis showed parameter that it is not a sensitive parameter |
| Protein binding to | AGP | Assumed, because basic compound (35) |
| Alpha-1-acid glycoprotein (AGP) KD (μM) | 5.81 | Predicted with Simcyp |
| AGP reference concentration (g/L) | 0.81 | Simcyp |
| **Absorption model (first order)** | | |
| Ka (h-1) | 1.4 | PopPK analysis (36) |
| Fa | 0.664 | PopPK analysis, calculated from F (36) |
| Peff (x 10-4 cm/s) | 2.44 | Calculated in Simcyp, based on doravirine Papp of 25*10-6 cm/s and verapamil Papp of 40.1*10-6 cm/s in LLC-PK1 cells (36) |
| **Distribution model** | | |
| Vss (L/kg) | 0.94 | Calculated in Simcyp with prediction method 2 and Kp scalar of 0.211 to simulate Vss as reported in PopPK analysis (36) |
| **Elimination model (enzyme kinetics)** | | |
| Total CYP3A4 CLint (μL/min/pmol CYP) | 0.026 | Yee et al, 2021 (10) |
| CLrenal | 0.556 | Yee et al, 2021 |

**Search query for PK studies with doravirine**

(((("doravirine"[Title/Abstract] AND ((("physiolog*"[Title] OR "mechanistic*"[Title]) AND ("kinetic*"[Title] OR "pharmacokinetic*"[Title]) AND ("model*"[Title] OR "simulat*"[Title])) OR "pbpk*"[Title/Abstract] OR "simcyp*"[Title/Abstract] OR "gastroplus*"[Title/Abstract] OR "PK-Sim"[Title/Abstract] OR "physiologically based pharmacokinetic model*"[Title/Abstract] OR "physiology based pharmacokinetic model*"[Title/Abstract])) NOT ("pesticides"[MeSH Terms] OR "pesticide*"[Title/Abstract] OR "insecticide*"[Title/Abstract] OR "herbicide*"[Title/Abstract] OR "fungicide*"[Title/Abstract] OR "rodenticide*"[Title/Abstract] OR "environmental pollution"[MeSH Terms] OR "metal*"[Title/Abstract] OR "environmental*"[Title/Abstract] OR "toxicant*"[Title/Abstract])) NOT (("animals"[MeSH Terms] NOT "humans"[MeSH Terms]) OR (("Mouse"[Title] OR "Mice"[Title] OR "Rat"[Title] OR "Rats"[Title]) NOT ("Man"[Title] OR "Men"[Title] OR "Woman"[Title] OR "Women"[Title] OR "human*"[Title] OR "adult*"[Title])))) NOT "Review"[Publication Type]) AND "English"[Language]

| ***Single dose studies*** | | | | | | | | | | | | |
| --- | --- | --- | --- | --- | --- | --- | --- | --- | --- | --- | --- | --- |
| **Study** | **Design** | **N** | **Age range (years)** | **Proportion of females** | **Health status** | **Co-medication** | **Study duration** | **Dose** | **Formulation** | **Fasted / Fed** | **Notes** | **Ref** |
| *Ankrom et al, A 2018* | Open-label, single-dose study comparing the PK of doravirine in HIV-negative subjects with severe renal impairment and healthy matched control subjects | 16 (8 with renal impairment, 8 healthy) | Healthy; mean (range); 60 (52-69) Renal impaired: 60.8 (51-69) | 3/8 and 2/8 resp | Healthy and with renal impairment (eGFR 104.8) | Not mentioned | 1 day | 100mg QD | Oral | Fasted (>8h) |  | *(31)* |
| *Khalilieh et al, 2016* | Open-label, single-dose study was conducted to evaluate the effect of moderate hepatic impairment on doravirine PK and to evaluate the safety and tolerability of doravirine in subjects with moderate hepatic insufficiency and in healthy subjects after single-dose administration | 8 healthy, 8 ,moderate hepatic impairment | Healthy (mean; range): 56 (44-63), hepatic 59 (54-64) | Healthy 2/8, Hepatic 2/8 | Healthy and hepatic impairment | CYP inhibitors or PGP substrate/inhibitors were excluded. Other drugs allowed if used for >1 month | 1 day | 100mg qd | Oral | Fasted (>8h) | Mild hepatic impairment 🡪 ChildPugh 7-9 | *(32)* |
| *Anderson et al 2019 (2) – DOR en TDF/3TC* | Anderson et al 2019 (1) – DOR en TDF. DOR single dose, single dose 3TC+ TDF + single dose DOR+TDF+3TC with 7 days of washout between single doses | 15 | 44 (23-56) | 8/15 | Healthy (although mean BMI 29.6) | TDF 300mg QD an/or 3TC 300mg |  | 100mg QD | Oral | Fasted (>8h) |  | (23) |
| *Khalilieh et al, 2019* | Open-label, 3-period, fixed-sequence drug interaction trial | 14 | 48 ± 10 | 6/14 | Healthy | Aluminium hydroxie, magnesium hydroxide and smethicone (one period) and after wash out pantozol | 30 days; 5 days one type of antiacid and 1 day of DOR. Washout in between | 100mg QD | Oral | Fasted (10h) |  | (37) |
|  | | | | | | | | | | | | |
| ***Multiple dose studies*** | | | | | | | | | | | | |
| **Study** | **Design** | **N** | **Age range (years)** | **Proportion of females** | **Health status** | **Co-medication** | **Study duration** | **Dose** | **Formulation / Infusion duration** | **Fasted / Fed** | **Notes** | **Ref** |
| *Matthew RP et al, A 2021* | Double-blind, placebo-controlled, randomized, fixed-sequence study to investigate PK and safety and tolerability of coadministration of DOR with islatravir | 14 | 33.5 (23-55) ; mean (range) | 1/7 | Healthy | Islatravir 2,25mg QD | 5 days | 100mg QD | Oral | Fasted (10h) |  | *(34)* |
| *Sterman FL et al, 2023* | Single center, open-label, observational switch trial that evaluated maintenance of virologic suppression among 20 HTE PWH with MDR virus, including a nested PK arm of 10 patients, who changed their antiretroviral regimen from RPV/FTC/TAF plus DTG to BIC/FTC/TAF plus DOR. | 10 | 65 (46-74), median (range) | 0 | Heavily treated people with HIV | Biktarvy and others, not specified | Follow up of 48 weeks | 100mg QD | Oral | Not mentioned | Characteristics of all participants in trial (n=20), PK subanalaysis was done in 10 patients, specifics not mentioned | (38) |
| *Lam E et al, 2020* | Phase I, prospective, open-label, two-period, fixed-sequence, drug-drug interaction study conducted in healthy volunteers | 11 | 46.4 (±9.9) | 1/11 | Healthy (although al obese, mean BMI 31) | INH and rifapentine | 21 weeks | 100mg BID | Oral | After a standardized meal |  | (39) |
| *Ankrom et al, 2019 (1) - Elbasvir- grazoprevir* | Phase 1, open-label, PK drug  interaction studies. | 12 | Median (range) – 29 (21.1 – 31.7) | 7/12 | Healthy | Elbasvir- grazoprevir | 5 days | 100mg QD | Oral | After a  moderate-fat breakfast, which was consumed after a fast of > 8 h. |  | (33) |
| *Ankrom et al, 2019 (2) – Ledipasvir – sofosbuvir* | “ | 14 | 36 (25-60) | 2/14 | Healthy | Ledipasvir – sofosbuvir | 5 days | 100mg QD | Oral | “ |  | (33) |

Table S2 Overview of doravirine studies to validate previously published PBPK model

**Table S3 Sensitivity analysis with data from participants who were shorter of longer than 12 months postpartum**

| PK parameter | Participants <12 months postpartum  N=5  GM (CV%)  or median (IQR) | Participants >12 months postpartum  N=3  GM (CV%) or median (IQR) | Ratio |
| --- | --- | --- | --- |
| *Plasma* | | | |
| AUC_inf_ (mg/L*h) | 19.38 (20) | 23.25 (46) | 0.83 |
| C_max_ (mg/L) | 1.03 (40) | 1.51 (12) | 0.68 |
| T_1/2_ (h) | 14.84 (24) | 10.79 (41) | 1.38 |
| *Breastmilk* | | | |
| AUC_inf_ (mg/L*h) | 5.06 (26) | 5.76 (23) | 0.88 |
| C_max_ (mg/L) | 0.30 (35) | 0.31 (40) | 0.97 |
| T_1/2_ (h) | 12.35 (20) | 10.36 (56) | 1.20 |
| MP ratio | 0.26 (23) | 0.25 (23) | 1.04 |
| DID (intake of 150mL/kg/day assumed) (mg/day) | 0.15 (0.15-0.16) | 0.31 (0.27-0.32) | 0.48 |
| RID (intake of 150mL/kg/day assumed) (%) | 2.25 (1.55-2.40) | 1.50 (1.42-2.37) | 1.50 |

Table S4A Sensitivity analysis with ranging breastmilk pH; geometric mean (CV%) of pharmacokinetic parameters in breastmilk and DID (mg) and RID

| Breastmilk pH | AUC_inf_ (mg/L*h) | C_max_ (mg/L) | MP ratio |
| --- | --- | --- | --- |
| 6.9 | 15.49 (52) | 1.02 (36) | 0,92 |
| 7.0 | 12.39 (52) | 0.82 (36) | 0,73 |
| 7.1 | 9.94 (52) | 0.66 (36) | 0,59 |
| 7.2 | 8.00 (52) | 0.53 (36) | 0,47 |
| 7.3 | 6.46 (52) | 0.43 (36) | 0,39 |
| 7.4 | 5.24 (52) | 0.35 (36) | 0,31 |

Table S4B Sensitivity analysis with ranging breastmilk creamatocrite values

| Breastmilk creamatocrite (%) | AUC_inf_ (mg/L*h) | C_max_ (mg/L) | MPratio |
| --- | --- | --- | --- |
| 2 | 6.37 (52) | 0.42 (36) | 0,38 |
| 4 | 6.46 (52) | 0.43 (36) | 0,38 |
| 6 | 6.55 (52) | 0.43 (36) | 0,39 |
| 8 | 6.65 (52) | 0.44 (36) | 0,39 |
| 10 | 6.74 (52) | 0.44 (36) | 0,40 |
